# A134 UES MANOMETRIC PARAMETERS IN ESOPHAGEAL MOTILITY DISORDERS

**DOI:** 10.1093/jcag/gwab049.133

**Published:** 2022-02-21

**Authors:** M Woo, D Randall, M Gupta, M Miles, D Y Li, Y Nasser, C N Andrews

**Affiliations:** University of Calgary, Calgary, AB, Canada

## Abstract

**Background:**

Upper esophageal sphincter (UES) function may be evaluated manometrically using a solid-state high-resolution manometry (HRM) system, which allows for the measurement of manometric parameters specific to the UES. While many of these parameters have yet to be validated for use in clinical practice, there is some suggestion that there may be an association between esophageal motility and UES function.

**Aims:**

We aimed to identify the relationship between UES manometric variables and high-resolution esophageal manometry (HREM) diagnoses.

**Methods:**

A retrospective analysis of HREM studies was performed between 2019 and 2021. Extraction of esophageal and UES manometric variables were performed. UES manometric values of interest included: mean basal pressure (mmHg), mean residual pressure (mmHg), relaxation time-to-nadir (ms), relaxation duration (ms), and recovery time (ms). Relationships between manometric diagnosis (Chicago Classification version 3) and UES manometric variables were explored. All values are expressed a medians and group means were compared with the non-parametric Mann-Whitney U test.

**Results:**

2119 symptomatic patients underwent HREM over the study period. Manometric diagnoses were achalasia (72 patients), esophagogastric junction outflow obstruction (286), absent contractility (108), distal esophageal spasm (53), jackhammer esophagus (32), and ineffective esophageal motility (694). 886 patients had no specific motility disorder; 643 of whom had ≤ 20% ineffective swallows and were considered symptomatic controls.

Patients with achalasia had significantly higher mean basal pressures (63.2 vs. 54.4, p = .001), mean residual pressure (3.8 vs. -1.9, p < .001), relaxation-time-to-nadir (182.0 vs. 142.0, p = .005), relaxation duration (820.5 vs. 708.0, p < .001) and recovery time (623.0 vs. 562, p < .001) compared to control patients. Among patients with achalasia, the presence of panesophageal pressurization correlated weakly with recovery time (R^2^ .3, p = .03). Patients with ineffective esophageal motility had significantly higher mean basal pressures (61.7 vs. 54.0, p < .001).

Among all patients, patients with incomplete bolus clearance (≥ 30%) had significantly higher UES mean basal pressure (58.9 vs. 54.6, p = .004), mean residual pressure (-.62 vs. -2, p < .001), relaxation duration (724.0 vs. 707.0, p = .014) and recovery time (580 vs. 558.0, p < .001).

**Conclusions:**

Patients with achalasia may have higher basal and residual UES pressures, and slower relaxation compared to patients with normal esophageal motility. This may reflect dynamic changes of the UES in response to obstruction at the esophagogastric junction. Elevated UES pressures are also seen in patients with ineffective esophageal motility, potentially reflecting a response to poor bolus clearance. More work needs to be done to validate these parameters in clinical practice.

**Funding Agencies:**

None

